# Diffuse Primary Angiosarcoma of the Pleura: A Case Report and Review of the Literature

**DOI:** 10.1155/2004/794907

**Published:** 2004-12-22

**Authors:** Jean-Emmanuel Kurtz, Sebastian Serra, Brigitte Duclos, François Brolly, Patrick Dufour, Jean-Pierre Bergerat

**Affiliations:** ^1^ Department of Hematology and Oncology, Hôpitaux Universitaires de Strasbourg, 1 Av Molière, Strasbourg 67098, France; ^2^ Department of Pneumology and CHG Saverne, Hôpitaux Universitaires de Strasbourg, 1 Av Molière, Saverne 67703, France

## Abstract

Primary pleural angiosarcoma is an extremely rare tumor. We report the case of a patient who presented with recurrent massive bilateral hemothoraxes. Although thoracoscopy was performed, biopsy samples of the pleura were inconclusive. The delayed onset of skin metastases led to the diagnosis of angiosarcoma, however the patient died from pleuropulmonary progression before treatment could be started. We review the literature of primary pleuropulmonary angiosarcoma and discuss its treatment modalities.


*Sarcoma*, December 2004, *VOL. 8, NO. 4, 103–106*

Taylor & Francis healthsciences

ORIGINAL ARTICLE

### Diffuse primary angiosarcoma of the pleura: A case report and review of the literature

JEAN-EMMANUEL KURTZ[1], SEBASTIAN SERRA[1], BRIGITTE DUCLOS[1], FRANÇOIS BROLLY[2], PATRICK DUFOUR[1] & JEAN-PIERRE BERGERAT[1]

[1]*Departments of Hematology and Oncology, Hôpitaux Universitaires de Strasbourg, 1 Av Molière, 67098 Strasbourg France and Pneumology and* [2]*CHG Saverne, 67703, Saverne, France*

**Abstract**
Primary pleural angiosarcoma is an extremely rare tumor. We report the case of a patient who presented with recurrent massive bilateral hemothoraxes. Although thoracoscopy was performed, biopsy samples of the pleura were inconclusive. The delayed onset of skin metastases led to the diagnosis of angiosarcoma, however the patient died from pleuropulmonary progression before treatment could be started. We review the literature of primary pleuropulmonary angiosarcoma and discuss its treatment modalities.

**Key words:** *angiosarcoma, pleura, hemothorax*

### Case report

A 61 year old caucasian male with no significant prior medical history was admitted for a spontaneous right hemothorax for which he underwent drainage and repeated blood transfusions. He rapidly relapsed, with the onset of a left hemothorax that was drained as well. CT-scan confirmed the hemothorax, but there was no evidence of mediastinal or lung abnormality (Figure 1). Bronchoscopy revealed no abnormality or intraluminal bleeding Angiography revealed hilar and peri-hilar hypervascularization as well as a vascular blush from thin collaterals of the right bronchial artery that was subsequently embolized with 500–700 µm embospheres (Boston Scientific). Abdominal ultrasonography was unremarkable. The patient underwent a diagnostic thoracoscopy that was inconclusive and only partial, in the setting of massive pleural bleeding and multiple pleural septa. One month later, a small hypervascularized lesion appeared on the left cheek, associated with gingival bleeding. Biopsy of the skin revealed malignant proliferation with large cells in mitosis, dissociated by hemorrhage (Fig. 2). Immunohistochemistry was negative for cytokeratin, desmin, leukocyte common antigen and carcinoembryonic antigen, but positive for CD31 and vimentin, leading to the diagnosis of an angiosarcoma, the patient was referred to our center, two months after the onset of the first symptoms. On clinical examination, he had a poor performance status (ECOG 3). He complained of fatigue, dyspnea, and had gingival bleeding. Cutaneous nodules had appeared on his back, consistent with secondary localizations of angiosarcoma. Blood count showed $52.10^3$/l leucocytes with $48.10^3$/l neutrophils, 88 g/l hemoglobin and $196.10^9$ platelets. Hemostasis was normal. Tumor markers were normal except for a slightly elevated CA125 at 59 U/ml, probably explained by the hemothorax. Blood chemistry was normal. Despite transfusions, and supportive care, the outcome was unfavorable and the patient died of acute respiratory failure before specific therapy could be started. An autopsy was performed, and confirmed the diagnosis of bilateral pulmonary angiosarcoma, with cutaneous and mucosal metastases.

### Discussion

Primary angiosarcoma of the chest wall or pleura is a rare condition and has a very poor prognosis.[1] Angiosarcomas represent 1 to 2% of soft tissue tumors.[2,3] It originates from endothelial cells from small blood vessels, and may affect a variety of

Correspondence to: Jean-Emmanuel Kurtz, Departments of Hematology and Oncology, Hôpitax Universitaires de Strasbourg, 1 Av Molière, Strasbourg, France. Tel.: +33 3 88 12 83 14; Fax: +33 3 88 12 76 81; E-mail: j-emmanuel.kurtz@chru-strasbourg.fr

1357-714X print/1369–1643 online © 2004 Taylor & Francis Ltd
DOI: 10.1080/1357-7140400003596

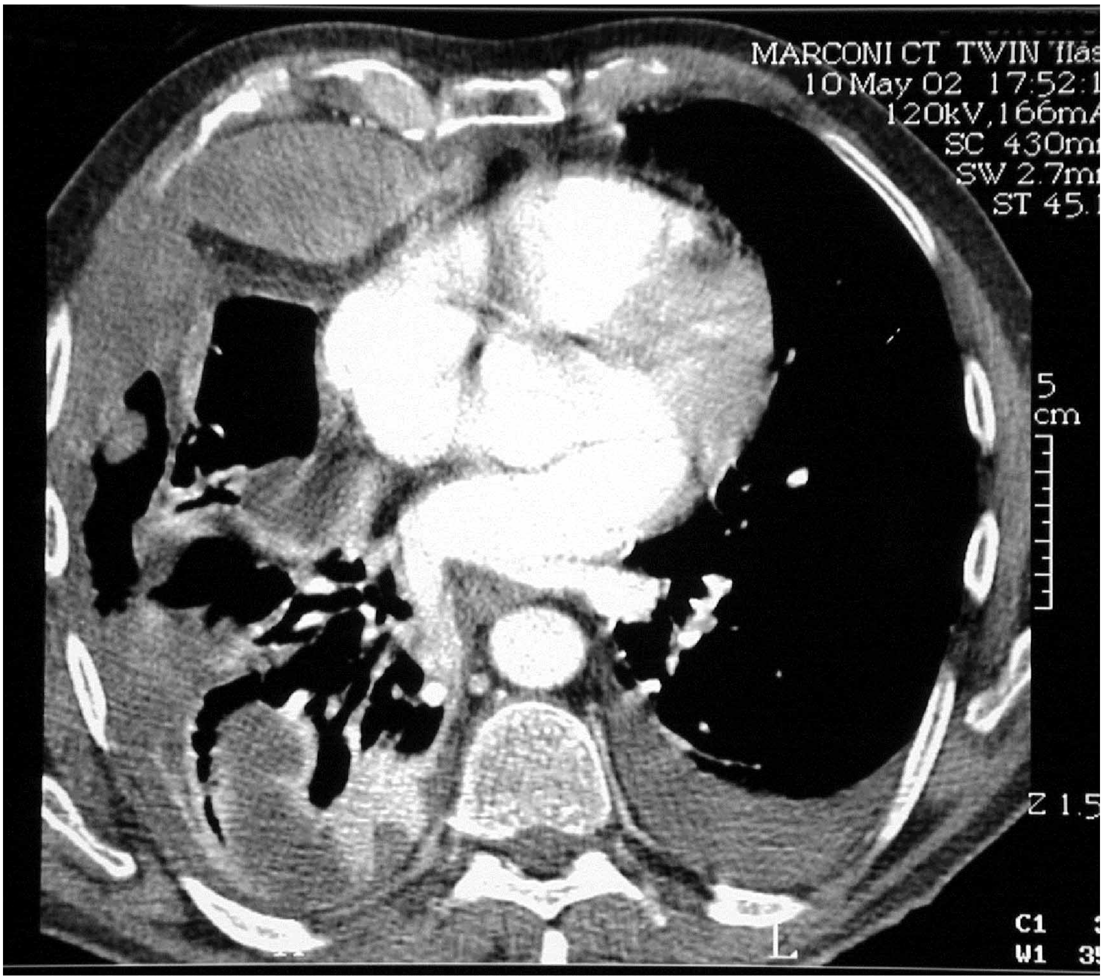

**Fig. 1.** *Thoracic CT-scan showing the bilateral hemothorax.*

organs, including the retroperitoneum, skeletal muscle, subcutis, liver, heart and breast.[4–6] Lung metastases from extrathoracic angiosarcoma have been described and are common in the natural history of angiosarcoma.[7] Patients with primary chest wall/lung angiosarcoma are often diagnosed in the setting of massive, recurrent hemothorax, although pain, chest wall mass, swelling or hemoptysis may represent the clinical picture.[1–3,8,9] However, as opposed to tumors arising from the chest wall, pleuropulmonary angiosarcoma patients usually present with a massive hemothorax, as in our case.[1,9,10] CT-scan findings include hemothorax, thickening of the pleura, or invasion of the chest wall. Pleural angiosarcoma has been described in the setting of chronic tuberculous pyothorax,[11] as well as a long-term consequence of radiotherapy,[12] or even without any prior history, e.g. "*de novo*" tumors.[1] Diagnosis may be difficult when biopsy samples are inconclusive, especially in the setting of recurrent massive bleeding within the pleura. In our case, the thoracoscopy was performed by a physician, without double lumen intubation. It is likely that such a procedure should be performed by a thoracic surgeon to facilitate the diagnosis. The positive staining for CD31, CD34 and vimentin, as well as the negativity for epithelial markers in the skin biopsy, together with morphologic criteria led to the diagnosis of primary pleural angiosarcoma metastatic to the skin and the oral mucosa. Given the history of recurrent hemothorax that occurred weeks before skin lesions appeared, it makes it unlikely that the patient had pleural metastases from cutaneous angiosarcoma. Post-mortem examination revealed a massive bilateral involvement of the pleura, invading the lung. Bronchio-alveloar hemorrhage was presumably the cause of the patient's death. Distant small skin and mucosal localizations were presumed metastatic, and the liver was free of either metastases or primary, as a case of liver angiosarcoma invading the pleura was reported to have induced a massive hemothorax.[13]

The outcome of pleural angiosarcoma is dismal.[4] Chemotherapy has little effect, and may relieve symptoms but can hardly be considered in patients with a poor performance status. Surgery is the best treatment for those patients who experience more circumscribed chest wall lesions, and is the key for long-term survival. Surgical debulking, pneumonectomy at the most, sometimes with rib excision in the case of chest wall tumors must be undertaken whenever possible.[1–3,9,14–16] Radiation

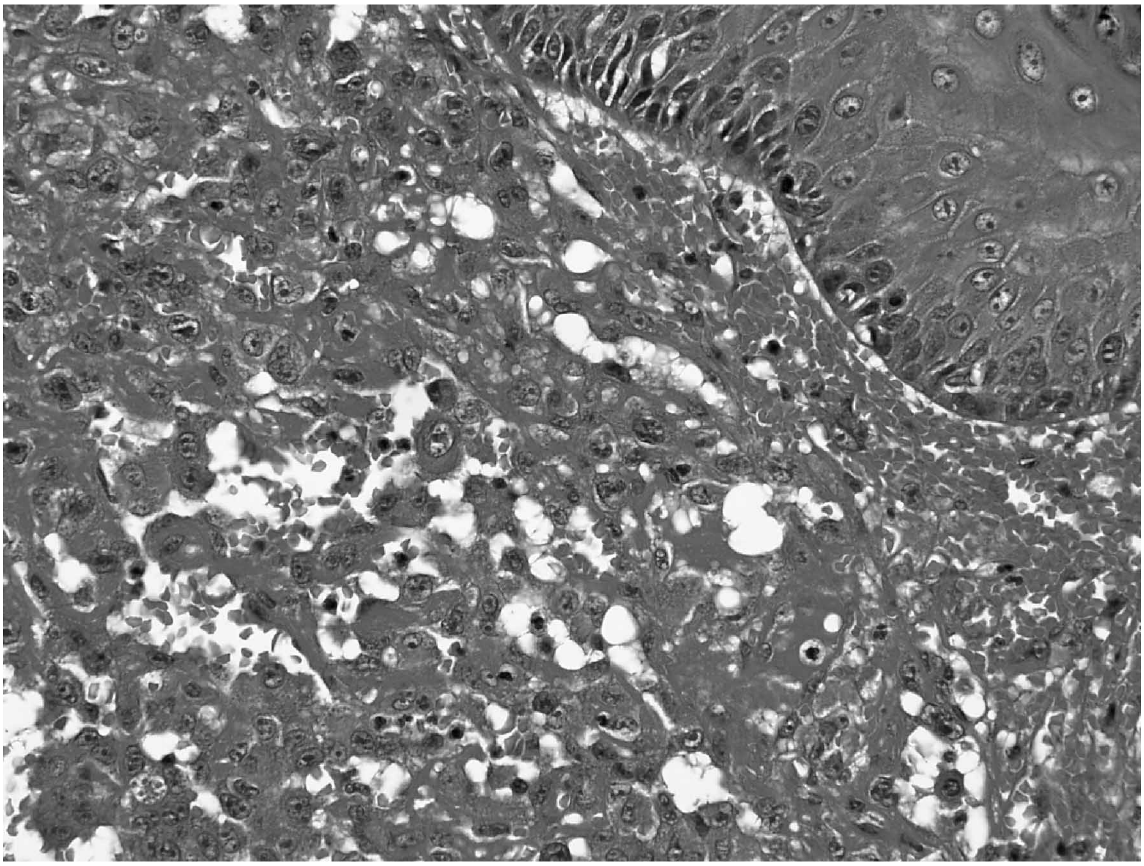

**Fig. 2.** *Skin biopsy (Hematoxillin-Eosin ×400). A hemorrhagic malignant proliferation consisting in large cells invades the superficial part of the dermis. Numerous mitoses are observed.*

therapy may have a role in the adjuvant setting for angiosarcoma[1,2,16] but it has little interest in patients with distant metastasis or diffuse pleural angiosarcoma, as in our case. Combined modality treatment has been reported in the neoadjuvant setting in a patient who secondarily underwent successful surgery of a left lung inferior lobe angiosarcoma.[8] However, neoadjuvant therapy might delay curative surgery and should not be recommended in current practice. Vascular embolization, as in our case, may decrease tumor feeding as well as pleural bleeding. It has also been performed prior to surgery, in order to decrease tumor vascularity.[3]

Massive hemothorax as the first symptom of an angiosarcoma has been reported in a few cases. Although pleural angiosarcoma is an extremely rare tumor, this dramatic clinical picture should not be misdiagnosed, in order to give the patients the best chance for surgical or medical therapy.

### References

1. Alexiou C, Clelland CA, Robinson D, Morgan WE. Primary angiosarcoma of the chest and pleura. *Eur J Cardio-Thoracic Surg* 1998; 14: 523–26.
2. Boulay V, Jeanfaivre T, Enon B, Saint-Andre JP, Tuchais E. Angiosarcoma of the thoracic wall revealed by hemothorax. *Rev Pneumol Clin* 1998; 54(2): 259–62.
3. Maziak DE, Shamji FM, Peterson R, Perkins DG. Angiosarcoma of the chest wall. *Ann Thorac Surg* 1999; 67: 839–41.
4. Patel AM, Ryu JH. Angiosarcoma in the lung. *Chest* 1993; 103(5): 1531–5.
5. Naka N, Ohsawa M, Tomita Y, Kanno H, Uchida A, Aozasa K. Angiosarcoma in Japan. A review of 99 cases. *Cancer* 1995; 75(4): 989–96.
6. Meis-Kindblom JM, Kindblom LG. Angiosarcoma of soft tissue: a study of 80 cases. *Am J Surg Pathol* 1998; 22(6): 683–97.
7. Bocklage T, Leslie K, Yousem S, Colby T. Extracutaneous angiosarcomas metastatic to the lungs: clinical and pathologic features of twenty-one cases. *Mod Pathol* 2001; 14(12): 1216–25.
8. Junge K, Toens C, Peiper C, Hermanns B, Schumpelick V. Primary angiosarcoma of the lung. *Chirurg* 2001; 72(8): 969–72.
9. Palvio DH, Paulsen SM, Henneberg EW. Primary angiosarcoma of the lung presenting as intractable hemoptysis. *Thorac Cardiovasc Surg* 1897; 35(2): 105–7.
10. Liu SF, Wu CC, Lai YF, Hsieh MJ. Massive hemoptysis and hemothorax caused by pleuropulmonary angiosarcoma. *Am J Emerg Med* 2002; 20(4): 374–5.
11. Aosaza K, Naka N, Tomita Y, Ohsawa M, Kanno H, Uchida A, Ono K. Angiosarcoma developing from chronic pyothorax. *Modern Pathol* 1994; 7(9): 906–11.
12. Case records of the Massachusetts General Hospital (Case 17-1987). *New Engl J Med* 1897; 316(17): 1075–83.

13. Wang YT, Wong SC, Thomas A. Locally invasive hepatic angiosarcoma: an unusual cause of massive hemothorax. *Thorax* 1983; 38(7): 558–9.
14. Okabayashi K, Hanagiri T, Noda Y, Ozaki S, Shiraishi T, Mitsudomi T, Okamura T, Hamada T, Shirakusa T. Angiosarcoma of the chest wall with a gastric metastasis. *Thorac Cardiovasc Surg* 1993; 41(5): 318–20.
15. Suzuki T, Suzuki S, Kitami A, Kamio Y, Hori G, Mitsuya T, Ota H. Angiuosarcoma of the chest wall. *Thorac Cardiovasc Surg* 1996; 44(4): 213–15.
16. Mark RJ, Poen JC, Tran LM, Fu YS, Juillard GF. Angiosarcoma. A report of 67 patients and a review of the literature. *Cancer* 1996; 77(11): 2400–6.
